# The distribution of lung cancer across sectors of society in the United Kingdom: a study using national primary care data

**DOI:** 10.1186/1471-2458-11-857

**Published:** 2011-11-10

**Authors:** Barbara Iyen-Omofoman, Richard B Hubbard, Chris JP Smith, Emily Sparks, Emma Bradley, Alison Bourke, Laila J Tata

**Affiliations:** 1 Division of Epidemiology and Public health, University of Nottingham, Clinical Sciences Building, City Hospital, Nottingham, NG5 1PB, UK; 2 Respiratory Biomedical Research Unit, University of Nottingham, UK; 3 Experian, Landmark House, Experian Way, NG2 Business Park, Nottingham, NG80 1ZZ, UK; 4 CSD Medical Research, 1 Canal Side Studios, 8-14 St Pancras way, London, NW1 OQG, UK

## Abstract

**Background:**

There is pressing need to diagnose lung cancer earlier in the United Kingdom (UK) and it is likely that research using computerised general practice records will help this process. Linkage of these records to area-level geo-demographic classifications may also facilitate case ascertainment for public health programmes, however, there have as yet been no extensive studies of data validity for such purposes.

**Methods:**

To first address the need for validation, we assessed the completeness and representativeness of lung cancer data from The Health Improvement Network (THIN) national primary care database by comparing incidence and survival between 2000 and 2009 with the UK National Cancer Registry and the National Lung Cancer Audit Database. Secondly, we explored the potential of a geo-demographic social marketing tool to facilitate disease ascertainment by using Experian's Mosaic Public Sector ™ classification, to identify detailed profiles of the sectors of society where lung cancer incidence was highest.

**Results:**

Overall incidence of lung cancer (41.4/100, 000 person-years, 95% confidence interval 40.6-42.1) and median survival (232 days) were similar to other national data; The incidence rate in THIN from 2003-2006 was found to be just over 93% of the national cancer registry rate. Incidence increased considerably with area-level deprivation measured by the Townsend Index and was highest in the North-West of England (65.1/100, 000 person-years). Wider variations in incidence were however identified using Mosaic classifications with the highest incidence in Mosaic Public Sector ™types 'Cared-for pensioners, ' 'Old people in flats' and 'Dignified dependency' (191.7, 174.2 and 117.1 per 100, 000 person-years respectively).

**Conclusions:**

Routine electronic data in THIN are a valid source of lung cancer information. Mosaic ™ identified greater incidence differentials than standard area-level measures and as such could be used as a tool for public health programmes to ascertain future cases more effectively.

## Background

More than two-thirds of people with lung cancer in the United Kingdom (UK) have advanced disease at the time of diagnosis when curative treatment can no longer be offered[1,2]. There exists socioeconomic variations in the incidence of lung cancer[3,4] and evidence from studies of other cancer screening services and treatments show unequal participation among different population sub-groups in screening services[5] as well as inequity in cancer treatment[6]. To increase earlier ascertainment of lung cancer and reduce lung cancer-related health inequalities, there is a public health need to enhance lung cancer awareness especially in sectors of society where lung cancer incidence is typically high, with a view to shorten the interval between symptoms and presentation to primary care. Computerised general practice records present a potentially useful source of data to understand the current pathway of lung cancer diagnosis in general practice as well as identify the societal distribution of lung cancer but their validity has yet to be established[7].

The Health Improvement Network (THIN) is a computerised longitudinal database of UK general practice records. It has been demonstrated to have high quality data[8] with a high degree of completeness and accuracy for records of cancer incidence[9] as well as other diagnoses [10-12]. THIN has not been fully exploited for lung cancer studies and its usefulness for lung cancer research will depend on its level of ascertainment and representativeness of lung cancer in the UK.

In addition to routine health information, patients' records in THIN have area-level information such as Strategic Health Authority (SHA) regions and the Townsend Index of multiple deprivation, which have been linked to patients' home postcodes. More recently, patients' records have also been linked to the Mosaic Public Sector ™ variable which is a consumer classification system originally designed by Experian to profile customers for the purpose of market research[13]. Compared with the well-known and commonly used Townsend Index[14] which measures the area-based level of material deprivation using four indicators: unemployment, car ownership, house ownership and overcrowding, Mosaic Public Sector ™ classifications take account of more granular characteristics of the population living at different UK postcodes and therefore allows a clearer identification of the characteristics and differing needs of people[15]. To date, Mosaic classification has been used to a limited extent for the targeting of population public health services to those most in need[16] and studies have usefully applied it to demonstrate social disparities in health-related behaviours such as heavy episodic drinking[17] and smoking prevalence[18].

The aims of this study were firstly, to assess the completeness and representativeness of overall and area-level lung cancer data in THIN and secondly, using Experian's Mosaic Public Sector ™ classification, identify the particular sectors of UK society where lung cancer incidence was highest. This could enable focused and targeted public health efforts to improve lung cancer awareness and care.

## Methods

The Health Improvement Network database is a computerised longitudinal database of general practice records that are collected regularly from each practice's clinical system without intervention to normal practice operation[19]. At the time of this study, THIN had data from 446 UK general practices with a total of 8.2 million people of which more than 3.2 million were actively registered and could be prospectively followed[19].

We identified all patients with a first recorded diagnosis of lung cancer from the 1st of January 2000 to the 28th of July 2009, which was the last date of data collection (Read codes for lung cancer diagnosis available on request) and then excluded all patients with codes for mesothelioma. Analysis was done using incident cases of lung cancer in order to obtain a measure of true survival of lung cancer patients and therefore avoid any survival bias that may arise with prevalent cases. To ensure that we had only incident cases, we included only patients who had been registered in the practice for at least 1 year prior to their first diagnosis of lung cancer. The denominator for incidence analyses included all patients in THIN general practices who had contributed data after the 1st of January 2000 and who had at least one year of data in the dataset. Incidence rates with 95% confidence intervals (CI) were calculated as the total number of new lung cancer cases per 100, 000 person-years at risk. To assess the completeness of lung cancer ascertainment in THIN general practices and whether this varied by different UK SHA regions, we calculated the THIN lung cancer incidence rates from 2006-2008 (period when lung cancer recording in our database was deemed most reliable) for each SHA and compared these with the 2003-2007 lung cancer rates recorded by the National Cancer Registry[20].

Overall incidence rates in the population were calculated for the study period (2000- 2009) and the results were stratified by calendar years (3-year periods), age (10-year age bands up to ≥ 90 years), sex, socioeconomic status and SHA region. Our measure of socioeconomic status was the Townsend Index of multiple deprivation in quintiles for each output area (approximately 150 households) using the 2001 census data[19]. We also calculated lung cancer incidence rates by Mosaic Public Sector ™ groups and types. Mosaic Public Sector ™ classification refines areas at a higher level than available deprivation markers by using data from 400 variables to classify all unique postcodes (approximately 15 to 20 households[21]) within the UK into 61 types, each type being a member of one of 11 groups (Additional file 1 Table S1). Classification is based on typical neighbourhood demographics, behaviour, consumer values, consumption patterns, lifestyle, education and social and health-related attitudes[22]. Because age and sex are used in part to derive the Mosaic Public Sector ™ classification, we did not adjust our Mosaic models for these covariates. Incidence rate ratios (IRR) between different population strata were obtained using multivariate Cox proportional hazards regression. We further analysed the incidence rate ratios using separate random effects Poisson regression models to adjust for any effects due to the variable reporting in general practices[23].

Lung cancer survival rates were calculated from the period of first recorded lung cancer diagnosis to death or the date of last data collection from the general practice. To further validate the lung cancer data in THIN, survival rates of lung cancer in THIN were compared with rates in the National Lung Cancer Audit database (LUCADA)[1], which is a good source of highly representative information on diagnosis and survival of lung cancer patients in NHS trusts throughout England, Wales and Scotland. Cox proportional hazards models were used to model survival data with age, sex and socioeconomic status to determine the relationship between these factors and lung cancer survival. The Cox proportional hazards assumption was assessed for each of the models by plotting the log minus log transformation of the Kaplan-Meier estimator of the survival function against time.

All analyses were done using STATA release SE11[24] and the study protocol was reviewed and approved in 2009 by the Cegedim Strategic Data Medical Research Scientific Review Committee.

## Results

We identified a total of 12, 135 incident cases of lung cancer recorded in THIN between the 1st of January 2000 and the 28th of July 2009. There were 7, 184 males and 4, 951 females comprising 59.2% and 40.8% of all lung cancer cases respectively. The median age at lung cancer diagnosis was 72.6 years (Inter-quartile range [IQR]: 64.5-79.0). The median age at lung cancer death was 73.8 years (IQR: 65.7-80.0).

### Lung cancer incidence

**Figure 1:**
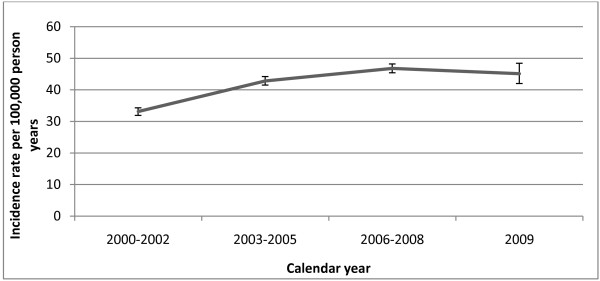
**Trend in incidence of lung cancer, 2000-2009**. Bars represent 95% confidence intervals.

The overall incidence of lung cancer in THIN for the whole study period from 2000 to 2009 was 41.4 per 100, 000 person-years (95% CI 40.6-42.1) (Table 1). There was an increase in the overall incidence of lung cancer by approximately 4% for every 3-year period (IRR 1.04, 95% CI 1.04-1.05) (Figure 1). The incidence rate in the 3-year period 2000-2002 was 33.1 per 100, 000 person years (95% CI 31.9-34.3). The incidence rate in 2003-2005 was 42.8 per 100, 000 person years (95% CI 41.5-44.2), incidence in 2006-2008 was 46.8 per 100, 000 person years (95% CI 45.4-48.2) and the incidence rate in 2009 was 45.1 per 100, 000 person years (95% CI 42.0-48.4).

**Table 1:** Overall incidence rates of lung cancer by age group and sex (2000-2009)

Age group (years)	Lung cancer events		100, 000 Person-yrs at risk		Rate/100, 000 person-years (95% CI)		
	**Male**	**Female**	**Male**	**Female**	**All**	**Male**	**Female**

0-40	30	29	75.6	72.4	0.4 (0.3-0.5)	0.4 (0.3-0.6)	0.4 (0.3-0.6)
40-50	168	147	22.1	21.3	7.3 (6.5-8.1)	7.6 (6.5-8.8)	6.9 (5.9-8.1)
50-60	793	574	19.3	18.9	35.7 (33.9-37.7)	41.0 (38.3-44.0)	30.3 (28.0-33.0)
60-70	1951	1285	14.5	14.9	110.0 (110-110)	134.4 (130-140)	86.3 (81.7-91.1)
70-80	2737	1781	9.6	11.7	212.0 (210-220)	286.2 (280-300)	151.6 (140-160)
80-90	1365	1029	3.9	7.0	219.4 (210-230)	348.1 (330-370)	147.2 (140-160)
> 90	139	105	0.5	1.5	120.2 (110-140)	283.7 (240-340)	68.2 (56.3-82.5)
All ages	7184	4951	145.5	147.8	41.4 (40.6-42.1)	49.4 (48.2-50.5)	33.5 (32.6-34.4)

Table 1 shows the variation in lung cancer incidence by sex and age. Incidence rates were 50% higher in males (49.4 per 100, 000 person-years, 95% CI 48.2-50.5) compared with females (33.5 per 100, 000 person-years, 95% CI 32.6-34.4) and increased with age, reaching a peak in the 80-90 year age-group in males and in the 70-80 year age-group in females.

Table 2 shows that the overall lung cancer incidence rate in THIN for all the SHAs between 2006-2008 was 46.8 per 100, 000 person-years accounting for 93.2% of the national cancer registry incidence rate of 50.2 per 100, 000 person-years. The highest rates of lung cancer in THIN were in the North-West of England followed by Scotland and the North-East of England and this pattern was similar over the entire study period from 2000-2009 (data for entire study period not shown). The lowest incidence rates were in London and the South-East coast. Comparing lung cancer incidence rates in THIN in the SHA regions over the 3 year period from 2006-2008 (when lung cancer incidence in THIN had increased from the initial stages of the study and reached a plateau) with national cancer registry rates, the rates in THIN and registry were comparable in 9 of the 13 SHAs. THIN incidence rates were higher than registry rates in the South-West of England but the rates were lower than registry rates in London, Northern Ireland and the West Midlands.

**Table 2:** Distribution of THIN lung cancer cases by UK Strategic Health Authority region

Strategic health authority (SHA)	Number of new cases of lung cancer in THIN 2006-2008	100, 000 person years at risk	THIN 2006-2008 lung cancer incidence rates/100, 000 person years (95% CI)	**UK national cancer registry age-standardised incidence rates of lung cancer (2003-2007)/100, 000 person yrs**[35]	Crude lung cancer incidence rate ratio (THIN compared to Registry rates)
East Midlands	172	4.1	41.7 (35.9-48.4)	47.1 (46.3-47.9)	0.89
East of England	331	7.6	43.5 (39.1-48.5)	40.6 (39.9-41.2)	1.07
London*	358	9.9	36.1 (32.5-40.0)	48.7 (48.0-49.4)	0.74
North East	211	3.3	63.6 (55.5-72.7)	68.2 (66.9-69.5)	0.93
North West	605	9.3	65.1 (60.1-70.5)	59.3 (58.6-60.1)	1.10
Northern Ireland*	146	3.8	38.8 (33.0-45.6)	49.2 (47.8-50.6)	0.79
Scotland	479	7.4	64.9 (59.4-71.0)	69.2 (68.3-70.1)	0.94
South Central	459	11.3	40.5 (36.9-44.4)	39.4 (38.6-40.2)	1.03
South East Coast	337	9.3	36.1 (32.5-40.2)	39.7 (39.0-40.5)	0.91
South West**	476	10.2	46.5 (42.5-50.8)	38.9 (38.3-39.6)	1.20
Wales	319	6.1	52.6 (47.2-58.7)	52.8 (51.8-53.9)	1.00
West Midlands*	372	9.3	39.8 (36.0-44.1)	46.5 (45.8-47.2)	0.86
Yorkshire & Humber	233	4.4	52.6 (46.2-59.8)	56.9 (56.0-57.7)	0.92

Overall	4498	96.2	46.8 (45.4-48.2)	50.2 (49.9-50.5)	0.93

* SHAs with lower incidence of lung cancer recorded in THIN compared to national cancer registry

** SHAs with higher incidence of lung cancer recorded in THIN compared to national cancer registry

(There is an overlap of the 95% confidence intervals in the incidence rates in the other 9 SHAs)

**Figure 2:**
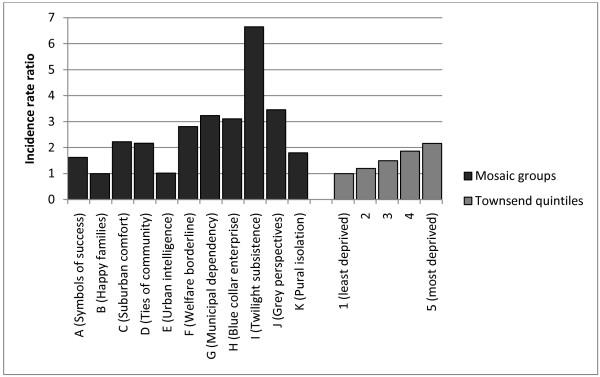
**Lung cancer incidence rate ratios by Mosaic Public Sector ™ groups and by Townsend quintiles (adjusted for age and sex)**. Reference groups (Mosaic group B; Townsend quintile 1).

We found a strong relationship between socioeconomic deprivation and lung cancer incidence (Table 3). Using the Townsend Index as a measure of area level deprivation, the highest lung cancer incidence rate of 61.5 per 100, 000 person-years (95% CI 59.1-64.1) in the most deprived Townsend quintile was over twice the incidence rate of 28.7 per 100, 000 person-years (95% CI 27.5-30.0) in the least deprived quintile. After adjusting for the effects of age, sex and general practice (Table 3 & Figure 2), there was an 11% increase in lung cancer incidence for every category increase in Townsend quintile (IRR 1.11, 95% CI 1.10-1.12) and the rate of lung cancer among people in the most deprived Townsend quintile was 2.2 times higher than the rate in the least deprived quintile (IRR 2.2, 95% CI 2.0-2.3).

**Table 3:** Overall incidence of lung cancer by Townsend Index quintiles and Mosaic Public Sector™ groups

	Lung ca events	Person-yrs at risk	Rate per 100, 000 p/y (95% CI)		Incidence rate ratios (95% CI) §	
		**Townsend index of deprivation**				

1 (least deprived)	2069	72.0		28.7 (27.5- 30.0)	1.00	
2	2243	61.4		36.5 (35.1- 38.1)	1.20(1.12-1.27)	
3	2439	58.1		42.0 (40.4- 43.7)	1.49(1.41-1.59)	
4	2653	51.4		51.7 (49.8- 53.7)	1.86(1.75-1.98)	
5 (most deprived)	2245	36.4		61.5 (59.1- 64.1)	2.16 (2.02-2.31)	
Missing	484	14.0		34.5 (31.5- 37.7)	1.29(1.14-1.46)	

		**Mosaic Public Sector ™ group**				

A (Symbols of success)	690	27.9		24.7 (23.0- 26.6)	1.38(1.24-1.54)	
B (Happy families)	613	34.9		17.6 (16.2- 19.0)	1.00	
C (Suburban comfort)	1700	46.7		36.4 (34.7- 38.1)	2.02(1.84-2.21)	
D (Ties of community)	1608	40.0		40.2 (38.2- 42.2)	2.27(2.07-2.50)	
E (Urban intelligence)	233	11.4		20.5 (18.0- 23.3)	1.28(1.10-1.49)	
F (Welfare borderline)	566	9.0		62.6 (57.6- 68.0)	3.65(3.26-4.09)	
G (Municipal dependency)	1008	15.4		65.5 (61.6- 70.0)	3.67(3.32-4.06)	
H (Blue collar enterprise)	1791	33.4		53.7 (51.2- 56.2)	2.99(2.73-3.28)	
I (Twilight subsistence)	866	6.7		129.3 (121.0- 138.2)	7.29(6.58-8.09)	
J (Grey perspectives)	1239	20.4		60.7 (57.4- 64.2)	3.44(3.13-3.79)	
K (Rural isolation)	425	14.1		30.0 (27.3- 33.0)	1.69(1.49-1.91)	
99 (Missing)	1394	33.3		41.9 (39.8- 44.2)	2.55(2.32-2.80)	

**§ **Townsend Index incidence rate ratios adjusted for age, sex and general practice

Mosaic Public Sector group incidence rate ratios adjusted for general practice

Compared with Townsend Index quintiles, there were wider variations in the incidence of lung cancer across Mosaic Public Sector ™ groups (Table 3 & Figure 2). The highest lung cancer incidence rate of 129.3 per 100, 000 person-years (95% CI 121.0-138.2) was found in Mosaic Public Sector ™ group I (Twilight subsistence). Mosaic Public Sector ™ groups F, G and J also had high rates of lung cancer incidence. After adjusting for the effects of general practice, the lung cancer incidence rate in Mosaic group I where incidence was highest, was 6.6 times higher when compared with the rate in Mosaic group B where the incidence of lung cancer was lowest (IRR 6.65, 95% CI 6.0-7.4)

**Figure 3:**
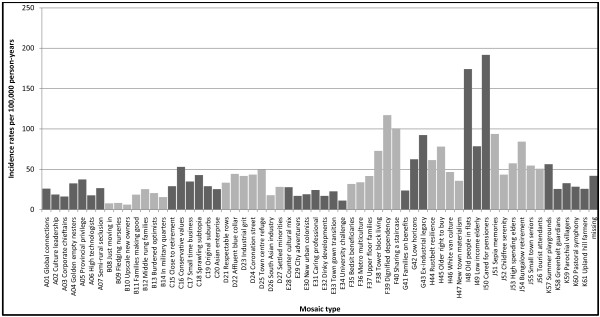
**Lung cancer incidence by Mosaic Public Sector ™ type**.

Analyses of the 61 Mosaic Public Sector ™ types (Figure 3) showed the highest lung cancer incidence rate of 191.7 per 100, 000 person-years (95% CI 173.8-211.5) in Mosaic Public Sector ™ type I50 (Cared for pensioners). The next highest incidence rate of 174.2 per 100, 000 person-years (95% CI 151.1-200.7) was found in Mosaic Public Sector ™ type I48 (Old people in flats). Lung cancer incidence was lowest for people in Mosaic Public Sector ™ type B10 (Upscale new owners) with a rate of 6.2 per 100, 000 person-years (95% CI 4.4-8.7). The incidence rate of lung cancer in Mosaic type I50 was 31.2 times higher (IRR 31.2, 95% CI 21.9-44.5) when compared to the rate in Mosaic type B10. Table 4 summarizes the typical characteristics of the Mosaic Public Sector ™ groups and types where lung cancer incidences were highest in the UK.

**Table 4:** Mosaic groups and types with the highest incidence of lung cancer

Mosaic groups	Characteristics
I Twilight subsistence	Older people living in social housing with high care needs
G Municipal dependency	Low income families living in estate based social housing
F Welfare borderline	People living in social housing with uncertain unemployment in deprived areas

**Mosaic types**	**Characteristics**

I50 Cared-for pensioners	Older people receiving care in homes or sheltered accommodation
I48 Old people in flats	Older people living in small council and housing association flats
F39 Dignified dependency	Low income couples and pensioners living in crowded apartments in high density social housing

### Lung cancer survival

Among the 12, 135 lung cancer cases studied, 8, 885 (73.2%) died during the study period. Six months after diagnosis, 57% of the cases were still alive; one year after, 37% of the cases were alive and five years after, only 11% of the cases were alive. The median survival of the cases was 232 days (IQR: 76-630 days). This was only slightly better than survival in LUCADA where the median survival was 203 days with a one year survival of 32%.

Male lung cancer patients died earlier than female patients with a median survival for males of 221 days (IQR: 72-580 days) compared with 251 days (IQR: 83-709 days) for females. The percentages of males alive at 6 months, 1 year and 5 years after diagnosis were 55%, 36% and 10% respectively. Survival for females on the other hand at 6 months, 1 year and 5 years were 59%, 40% and 12% respectively. Survival for patients in THIN was better than survival in the cancer registry[25], where the one year lung cancer survival was 27% for men and 30% for women. After adjusting for the effect of age at diagnosis, male lung cancer patients in THIN had 11% worse survival than female lung cancer patients (Hazards ratio for death - 1.11, 95% CI 1.06 to 1.16). Table 5 shows that lung cancer survival worsened with increasing age at diagnosis. Using the Townsend index deprivation quintile as a measure of socioeconomic status, survival did not differ across socioeconomic groups.

**Table 5:** Survival of lung cancer patients by age at diagnosis and Townsend quintiles

	Median survival in days (IQR)	6 months survival	1-year survival	5-year survival	Unadjusted hazards ratio	95% CI	p-value
**Age at diagnosis (years)**							
< 40	457 (248- .)	85%	52%	31%	1.00	-	-
40-50	341 (148-1150)	70%	48%	17%	1.35	0.92-1.97	0.126
50-60	287 (116-830)	65%	42%	15%	1.54	1.08-2.20	0.018
60-70	274 (85-736)	61%	42%	13%	1.68	1.18-2.40	0.004
70-80	218 (72-604)	55%	36%	9%	1.94	1.36-2.77	< 0.001
80-90	164 (54-443)	47%	29%	6%	2.41	1.69-3.45	< 0.001
> 90	147 (46-403)	40%	26%	-	2.72	1.85-4.01	< 0.001

**Townsend score (quintiles)**							
1	223 (78-593)	56%	37%	9.7%	1.00	-	-
2	232 (79-640)	57%	36%	10%	0.98	0.91-1.05	0.53
3	224 (67-587)	56%	36%	9.9%	1.03	0.96-1.10	0.46
4	242 (76-666)	58%	39%	12%	0.94	0.88-1.01	0.10
5	221 (72-608)	55%	37%	10%	1.01	0.94-1.09	0.82
missing	296 (116-1032)	64%	44%	18%	0.78	0.68-0.88	< 0.001

## Discussion

The overall incidence rate of lung cancer recorded in THIN general practices was 41.4 per 100, 000 person-years between 2000 and 2009, however incidence from 2000-2002 was lower than in the latter periods of the study. This supports findings from a previous study which showed that the observed recording rates of pancreatic, colorectal and lung cancers in THIN prior to 2004 were lower than expected based on the national cancer registry data but increased to approximately 80% of registry rates after 2004[9]. It has been suggested that a large increase in the recruitment of general practices to THIN in 2003 associated with receipt of training in data entry, experience in using the Vision software, and the institution of cancer quality improvement measures by the national Health Service in 2003 may have all contributed to the increase in recording of these cancers[9]. The introduction of the Quality and Outcomes Framework (QOF)[26] in 2004 which encourages general practitioners to record all new cases of cancer may also partly explain the increase in cancer recording in THIN. After comparing the lung cancer incidence rate in THIN with incidence rate recorded by the national cancer registry[20], our study confirms that THIN captures a significantly higher proportion of lung cancer incidence in more recent years.

### Lung cancer Incidence

There are two reliable national lung cancer databases in the UK against which THIN data can be compared to assess its completeness and representativeness. These are the National Lung Cancer Audit database (LUCADA)[1] which has been shown to be highly representative of people with lung cancer in England[27]; and the national cancer registry data reported by the Office of National Statistics (ONS)[28] which is a good source of information on lung cancer incidence. Data reported by the ONS are systematically collected from all regional cancer registries in England, Wales, Scotland and Northern Ireland.

Reassuringly, the sex distribution of lung cancer cases in THIN, the median age at diagnosis and at death, and the increasing incidence with greater socioeconomic deprivation were all comparable to findings from LUCADA[27]. Comparison of the lung cancer incidence rate in THIN with the incidence rate reported by the national cancer registry[20], showed the incidence rate in THIN to be over 93% of the cancer registry incidence rate. Geographical variations in lung cancer incidence in THIN were also mostly similar to registry data. The highest incidence rates were in the North-West of England, North-East of England and Scotland while the South East Coast and London had the lowest incidence. Cancer registry data however, shows incidence in London to be exceptionally high compared to other SHA regions in southern England. This is in contrast to THIN where the lowest incidence of lung cancer was in London, which may be due to THIN's over recruitment of practices covering slightly more affluent areas[29,30]. The population of THIN also has an over-representation of practices from the South-East of England where incidence rates are among the lowest so it is therefore unsurprising that the crude overall lung cancer incidence in THIN is marginally lower than the incidence rates based on registry data. The difference between THIN and registry incidence rates may also be partly attributed to the fact that about 6.8% of cases included in the UK cancer registries are from death certificates only[31].

### Societal distribution of lung cancer

The association we found between lung cancer incidence and greater socioeconomic deprivation was independent of age, sex and general practice and is consistent with findings from other studies[3,4]. Variations in lung cancer incidence were however, more marked in the Mosaic groups and types than in Townsend deprivation quintiles. Mosaic Public Sector ™ segmentation classifies UK households and postcodes into several lifestyle groups and types based on finer characteristics which enabled us to identify much higher incidence rates of lung cancer in specific sectors of society. Mosaic Public Sector ™ types I50 (Cared for pensioners), I48 (Old people in flats) and F39 (Dignified dependency) had the highest lung cancer incidence rates and this was unsurprising considering the fact that these Mosaic Public Sector ™ types are characterised mostly by older people who have poor levels of education, are mostly reliant on state benefits and live relatively less healthy lifestyles including above average smoking rates.

Mosaic classification is done at the household as well as the postcode level and although about half (54%) of the data used for Mosaic profiling are sourced from the 2001 census, the other 46% are derived from sources such as the Experian Lifestyle Survey, consumer credit databases, the electoral roll, shareholder registers, Land registry data, Council Tax information, the Hospital Episode Statistics, the British Crime Survey, Expenditure and Food Survey and other sources[13]. Mosaic profiling is therefore based on an exchange of information which enhances a deeper understanding of the characteristics of people in the various groups and types[15] unlike the Townsend Index which uses a less complex classification of postcodes based on measures of socioeconomic deprivation from census data[14]. To accurately target public health resources and develop tailored public health campaigns and interventions, the differing needs of deprived populations have to be identified and understood and in this regard, Mosaic classification is particularly valuable.

### Lung cancer survival

Median survival for people with lung cancer in THIN was only slightly better than survival in LUCADA[27]. The survival estimates in THIN and LUCADA were marginally higher when compared with survival in the cancer registry[25] and most likely reflect the different methods of case ascertainment[32]; in particular, the registry ascertains cases with a diagnosis of lung cancer only on a death certificate whilst these cases, having no supporting clinical data prior to death, may not have been recorded in THIN nor LUCADA.

Socioeconomic deprivation did not affect survival of people with lung cancer in THIN and this is consistent with the findings from LUCADA[27]. This lack of association may reflect the dismal prognosis of lung cancer in general and the lack of effective treatments for most people with lung cancer.

A major strength of this study is that Experian's Mosaic Public Sector ™ classification tool provides a finer and more detailed classification of the UK population than any other socio-demographic classification markers such as Townsend deprivation index[14] and therefore allows programs and interventions to be tailored to the specific needs of the population.

Potential limitations of using routine general practice data for research is that detailed diagnostic criteria for medical conditions may vary between practices and between doctors in the same practice[33] and analyses using these data assume the best diagnostic formulation without taking account variations in the perception of morbidity. The diagnosis of lung cancer is however made following investigations carried out by chest physicians in secondary care[34]. It is therefore unlikely that differences in GPs diagnostic criteria or perception of the disease would have had a large impact on the records of lung cancer in this analysis.

## Conclusion

Our analyses have shown that general practice data from THIN are representative of lung cancer in the UK and capture the vast majority of cases from cancer registries. UK general practice data are thus a potentially valuable tool for lung cancer research as they are the only source of detailed prospectively collected health information available at a population level both before and after lung cancer diagnosis. Linkage of patients' records to Experian's Mosaic Public Sector ™ classification has also provided us with a more refined knowledge of the sectors of society where lung cancer incidence is highest in the UK. As such, Mosaic could be used outside general practice as an important tool to reduce lung cancer-related health inequalities by enabling tailored public health campaigns and interventions to be more precisely and thus effectively targeted geographically to specific lifestyle groups in society.

## Competing interests

Barbara Iyen-Omofoman, Laila J Tata, Chris JP Smith and Richard B Hubbard declare that they have no competing interests. Emily Sparks and Emma Bradley work for Experian, Nottingham, UK and Alison Bourke works for Cegedim Strategic Data Medical Research, London, UK. Although these organizations have provided data for this study, this did not influence the objectives or outcome of this study, which was designed and conducted at the University of Nottingham.

## Authors' contributions

RH, LT, BI-O, AB, ES and EB conceived the idea for and designed this study. AB provided the THIN data while ES and EB provided the MOSAIC data. CS extracted the THIN data and ensured its accuracy. BI-O performed the statistical analysis and wrote the first draft of the manuscript. All authors critically revised and approved the final manuscript

## Pre-publication history

The pre-publication history for this paper can be accessed here:

http://www.biomedcentral.com/1471-2458/11/857/prepub

## Supplementary Material

Additional file 1 **Mosaic Public Sector ™ groups and types**. Click here for file
