# Supplementary material for: The distribution of lung cancer across sectors of society in the United Kingdom: a study using national primary care data
Source: BMC Public Health. 2011 Nov 10;11:857. doi: 10.1186/1471-2458-11-857 (PMC3282683; doi:10.1186/1471-2458-11-857)
Supplement: Additional file 1 — Mosaic Public Sector ™ groups and types. [file 1471-2458-11-857-S1.PDF]

| Code | Mosaic Public Sector™ group | Code | Mosaic Public Sector™ type |
|---|---|---|---|
| A (Symbols of success) | Career professionals living in sought after locations | A01 | Financially secure people living in smart flats in cosmopolitan inner city locations |
| | | A02 | Highly educated senior professionals, many working in the media, politics and law |
| | | A03 | Successful managers living in very large houses in outer suburban locations |
| | | A04 | Financially secure couples, many close to retirement, living in sought after suburbs |
| | | A05 | Senior professionals and managers living in the suburbs of major regional centres |
| | | A06 | Successful, high earning couples with new jobs in areas of growing high tech employment |
| | | A07 | Well paid executives living in individually designed homes in rural environments |
| B (Happy families) | Younger families living in newer homes | B08 | Families and singles living in developments built since 2001 |
| | | B09 | Well qualified couples typically starting a family on a recently built private estate |
| | | B10 | Financially better off families living in relatively spacious modern private estates |
| | | B11 | Dual income families on intermediate incomes living on modern estates |
| | | B12 | Middle income families with children living in estates of modern private homes |
| | | B13 | First generation owner occupiers, many with large amounts of consumer debt |
| | | B14 | Military personnel living in purpose built accommodation |
| C (Suburban comfort) | Older families living in suburbia | C15 | Senior white collar workers many on the verge of a financially secure retirement |
| | | C16 | Low density private estates, now with self reliant couples approaching retirement |
| | | C17 | Small business proprietors living in low density estates in smaller communities |
| | | C18 | Inter war suburbs many with less strong cohesion than they originally had |
| | | C19 | Attractive older suburbs, typically occupied by families but with increasing singles and childless couples |
| | | C20 | Suburbs sought after by the more successful members of the Asian community |
| D (Ties of community) | Close-knit, inner city and manufacturing town communities | D21 | Mixed communities of urban residents living in well built early 20th century housing |
| | | D22 | Comfortably off manual workers living in spacious but inexpensive private houses |
| | | D23 | Owners of affordable terraces built to house 19th century heavy industrial workers |
| | | D24 | Low income families living in cramped Victorian terraced housing in inner city locations |
| | | D25 | Centres of small market towns and resorts containing many hostels and refuges |
| | | D26 | Communities of lowly paid factory workers, many of them of South Asian descent |
| | | D27 | Multi-cultural inner city terraces attracting second generation settlers from diverse communities |
| E (Urban intelligence) | Educated, young, single people living in areas of transient populations | E28 | Neighbourhoods with transient singles living in multiply occupied large old houses |
| | | E29 | Economically successful singles, many living in privately rented inner city flats |
| | | E30 | Young professionals and their families who have gentrified terraces in pre 1914 suburbs |
| | | E31 | Well educated singles and childless couples colonising inner areas of provincial cities |
| | | E32 | Singles and childless couples in small units in newly built private estates |
| | | E33 | Older neighbourhoods increasingly taken over by short term student renters |
| | | E34 | Halls of residence and other buildings occupied mostly by students |

| | | | |
|---|---|---|---|
| F (Welfare borderline) | People living in social housing with uncertain employment in deprived areas | F35 | Young people renting hard to let social housing often in disadvantaged inner city locations |
| | | F36 | High density social housing, mostly in inner London, with high levels of diversity |
| | | F37 | Young families living in upper floors of social housing |
| | | F38 | Singles, childless couples and older people living in high rise social housing |
| | | F39 | Older people living in crowded apartments in high density social housing |
| | | F40 | Older tenements of small private flats often occupied by highly disadvantaged individuals |
| G (Municipal dependency) | Low income families living in estate based social housing | G41 | Families, many single parent, in deprived social housing on the edge of regional centres |
| | | G42 | Families with school age children, living in very large social housing estates on the outskirts of provincial cities |
| | | G43 | Older people, many in poor health from work in heavy industry, in low rise social housing |
| H (Blue collar enterprise) | Upwardly mobile families living in homes bought from social landlords | H44 | Manual workers, many close to retirement, in low rise houses in ex-manufacturing towns |
| | | H45 | Older couples, mostly in small towns, who now own houses once rented from the council |
| | | H46 | Residents in 1930s and 1950s council estates, typically in London, now mostly owner occupiers |
| | | H47 | Social housing, typically in 'new towns', with good job opportunities for the poorly qualified |
| I (Twilight subsistence) | Older people living in social housing with high care needs | I48 | Older people living in small council and housing association flats |
| | | I49 | Low income older couples renting low rise social housing in industrial regions |
| | | I50 | Older people receiving care in homes or sheltered accommodation |
| J (Grey perspectives) | Independent older people with relatively active lifestyles | J51 | Very elderly people, many financially secure, living in privately owned retirement flats |
| | | J52 | Better off older people, singles and childless couples in developments of private flats |
| | | J53 | Financially secure and physically active older people, many retired to semi rural locations |
| | | J54 | Older couples, independent but on limited incomes, living in bungalows by the sea |
| | | J55 | Older people preferring to live in familiar surroundings in small market towns |
| | | J56 | Neighbourhoods with retired people and transient singles working in the holiday industry |
| K (Rural isolation) | People living in rural areas far from urbanisation | K57 | Communities of retired people and second homers in areas of high environmental quality |
| | | K58 | Well off commuters and retired people living in attractive country villages |
| | | K59 | Country people living in still agriculturally active villages, mostly in lowland locations |
| | | K60 | Smallholders and self employed farmers, living beyond the reach of urban commuters |
| | | K61 | Low income farmers struggling on thin soils in isolated upland locations |
